# Rare presentation of metastatic renal cell carcinoma to thyroid gland: A case report

**DOI:** 10.1016/j.amsu.2020.06.021

**Published:** 2020-06-20

**Authors:** Ammar H. Habibullah, Sherif K. Abdelmonim, Ahmad Aldajani, Mohannad Rajab, Mohammad Alessa, Haddad Alkaf

**Affiliations:** a Department of Otolaryngology Head and Neck Surgery, King Abdullah Medical City, Makkah, Saudi Arabia; b Department of Otolaryngology Head and Neck Surgery, University of Jeddah, Jeddah, Saudi Arabia; c Department of Otolaryngology Head and Neck Surgery, Ministry of Health, Makkah, Saudi Arabia

**Keywords:** Thyroid nodule, Renal cell carcinoma, Thyroid metastasis

## Abstract

**Introduction:**

Renal cell carcinoma is known to cause metastasis to unusual sites, but metastasis to the thyroid gland is a rare occurrence, not only that, but 20 years after complete recovery is almost nonexistent.

**Case presentation:**

We are presenting here a case of 79-year-old female who presented to us for asymptomatic progressive thyroid nodule. She had history of right nephrectomy for renal cell carcinoma 20 years ago. Ultrasound guided fine needle aspiration biopsy (FNAB) of the thyroid was non-diagnostic. Total thyroidectomy of the patient was performed, and histopathological evaluation of the specimen revealed the swelling to be of metastatic in nature secondary to renal cell carcinoma.

**Discussion:**

Despite thyroid metastasis being rare, FNAB can prove to be useful tool for diagnosis of thyroid tumors and metastases and can be inconclusive in some cases. Therefore, immunohistochemistry can prove beneficial in diagnosis such cases.

**Conclusion:**

The diagnosis of renal cell carcinoma metastasis is made after immunohistochemical evaluation of the thyroidectomy specimen with thyroidectomy proving to be the modality of treatment for such cases with no further radiation therapy and a yearly follow up plan to screen for other lesions.

## Highlights

• Renal cell carcinoma is one of the most common malignany to metastasize to thyroid.
• Metastatic etiology should always be considered in thyroid nodule patient with history of renal cell carcinoma.
• Diagnosis is mainly by immunohistochemical analysis.
• Surgery has a significant mortality benefit.

## 1 Introduction

Overall, 3% of total malignant burden in adults is caused by renal tumors [1] with the most common histological type of renal tumor to be renal cell carcinoma [1]. Renal cell carcinomas are known for their unpredictable clinical course and are known to cause distance and late metastasis to unusual locations [2,3]. An autopsy series showed that renal cell carcinoma leads to metastasis to thyroid gland in 4%–5% cases, but presence of clinically significant metastasis to the thyroid is a rare clinical scenario [3]. And as thyroid tumors are mostly considered to be of primary origin, their characterization from secondary is a medical challenge. This case has been reported in accordance with SCARE 2018 Criteria [4].

## 2 Case presentation

A 79 year-old-female known case of Diabetes Mellitus-2, Hypertension, Ischemic Heart Disease and Atrial Fibrillation presented to our clinic complaining of progressive, painless central neck swelling for a duration of 4 months. Furthermore, there was no history of dysphagia, odynophagia, dyspnoea, hoarseness, stridor, aspiration or fever. Regarding her family history, there is no history of thyroid cancer nor malignancy or previous radiation exposure and no psychological or relevant genetic history. Meanwhile, her drug history includes Metformin, Amlodipine, Bisoprolol, Isosorbide Dinitrate and Apixaban. Moreover, there were no clinical symptoms relating to hyperthyroidism or hypothyroidism. Lastly, surgical history of right sided Nephrectomy due to Renal Cell Carcinoma was performed 20 years back with complete recovery.

On clinical neck examination, a firm 6 × 5 cm, non-tender central neck mass was noted with no overlying skin abnormalities and no palpable lymph nodes. A further thyroid workup was carried showed normal thyroid function tests while ultrasound showed multiple bilateral thyroid nodules with largest being 2.8cm on the left thyroid lobe. Therefore, a decision for a Fine Needle Aspiration (FNA) Biopsy was made to determine the swelling pathology but the result was non-diagnostic (Bethesda type 1). Subsequent neck CT showed thyroid gland enlargement with bilateral heterogeneous lesions and some calcifications in the left lobe. The left thyroid lobe measured 4.5 × 5.5 × 11 cm with retrosternal extension while the airway was narrowed and displaced towards the right side with no signs of obstruction. Meanwhile, there were no significantly enlarged cervical lymph nodes and all the major blood vessels were patent. Such imaging result with the progressiveness nature of the mass warranted to not repeat the FNA and proceed with surgery. Therefore, a fibre-optic nasopharyngoscopy was performed pre-operatively showed bilateral mobile vocal folds with unremarkable otolaryngology examination.

Regarding surgery, the patient underwent a total thyroidectomy with no intra-operative complications by an experienced head & neck surgeon of more than 15 years. A drain was placed, and the patient was shifted to surgical ICU for observation. During her first day post-operatively, she was extubated, and the drain was removed. Subsequently. The patient was shifted to the surgical ward on the third postoperative day and was discharged on the fourth postoperative day in a stable condition.

**Fig. 1:**
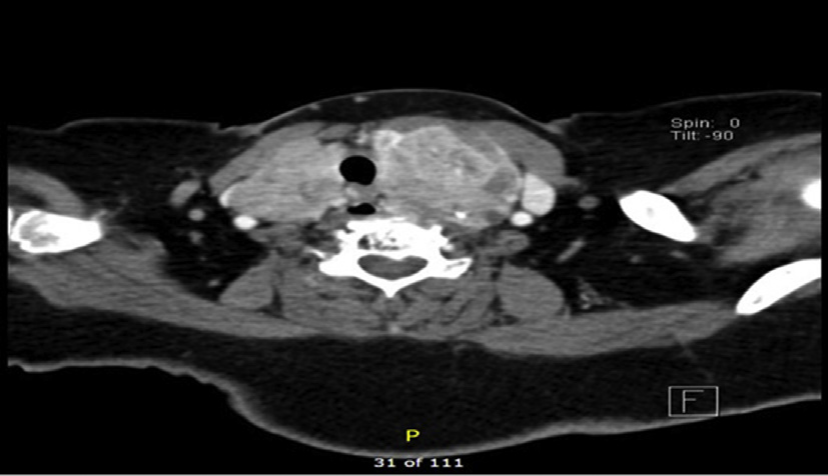
CT with contrast, Axial view demonstrating heterogeneous enlarged left thyroid lobe shifting the airway to the right side.

**Fig. 2:**
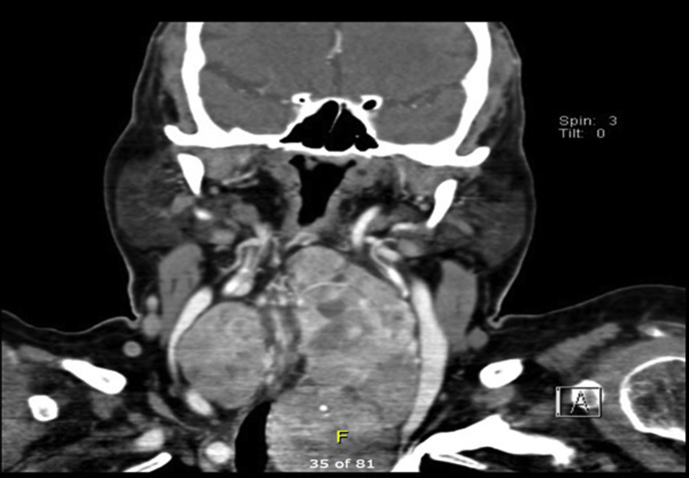
CT with contrast, Coronal view demonstrating heterogeneous left thyroid lobe enlargement with retrosternal extension.

Regarding her histopathology report, the thyroid specimen showed infiltration of thyroid tissue on both lobes with neoplastic nodules with largest measuring 3 × 2.5 × 2 cm on the left lobe, composed of large clear cells. On the microscopic description, it was noted by the pathologist that sections from both lobes revealed infiltration of thyroid tissue by neoplastic nodules composed of large clear cells. These cells are polygonal exhibiting clear cytoplasm, distinct cell boundaries and small rounded to slightly irregular nuclei with inconspicuous nucleoli. The cells are mainly arranged in groups and alveoli separated by network of thin walled vasculature. Immunohistochemical stains showed the tumour cells to be positive for Renal Cell Carcinoma (RCC), Vimentin (patchy) and Epithelial Membrane Antigen (EMA) while negative for Thyroglobulin and Thyroid Transcription Factor 1 (TTF1). Afterwards, the case was discussed in the tumour board and planned for urology consultation and follow up only after clearance the suspicion of occurrence of renal carcinoma of the other kidney with negative scans. As radioactive iodine treatment was not indicated, the patient was seen in the clinic after one week, one month, and three months' intervals and she was found to be asymptomatic with no significant complaints and no signs of other metastases. The patient was compliant throughout the course of treatment, but was in shock when she learned that the cause was her renal cancer that was treated 20 years ago (see Fig. 1, Fig. 2).

## 3 Discussion

Renal cell carinomas are known to be notorious in nature. They are famed for their unpredictabel clinical course and they can show late and distant recurrences [5,6]. The most common route for metastasis of renal malignancy is hematogenous and secondaries most commonly involving the lung, liver, bone, adrean galnd and brain. Thyroid gland is not a usual site for renal cell carcinoma metastasis but renal cell carcinom does remains one of the most common malignany to metastasize to thyroid despite the rarity of occurance [[7], [8], [9]]. In some of the rare instances, renal cell carinoma can also present as a thyroid nodule which can be easily misdiagnosed as primaly thyroid neoplasm. Especially the majority of metastases reported to the thyroid gland are metachronous with a prolonged average time for development of 9.4 years after resection of the primary renal cell carcinoma [7].

Although autopst studeis claim a higher percentage for thyroid secondaries, clinically significant thyroid metastasis is a rare occurrence [6,10] with majority of cases with secondaries to the thyroid gland not having any clinical symptom at the time of presentation [11]. Meanwhile, imaging studies of the thyroid like ultrasound and CT scans are usually non-diagnostic and cannot differentiate primary thyroid malignancy from metastasis [11,12]. However, FNA biopsy is considered the most logical next step considering its low cost and low invasiveness as it is an effective tool for the diagnosis of thyroid metastases. However, sometimes it becomes difficult to differentiate a thyroid tumor from a secondary and as chund et al. review demonstrated that FNAB presents with a high false negative rate in the range of 28.7% [2]. Hence, metastasis to the thyroid gland cannot be ruled out even in cases of inconclusive and negative for malignant cells [2].

Secondries to thyroid gland are most commonly recognised by immunohistochemical analysis of the thyroidectomy specimen. Diagnosis of a secondary renal cell carcinoma is confirmed by having a positive immunohistochemical results for vimentin and CD10 [11,12] and the specimen should tbe negative for TTF-1, thyroglobulin and calcitonin [5].

A study done by Machens et al., clearly showed that there is significant mortality benefit if surgical approach is chosen in the management of secondaries of the thyroid gland [13]. Therefore, surgical resection of the thyroid is the preffered approach in patients in whom only site of secondary metastasis is the thyroid gland [6]. Patients with disseminated disease have more grave prognosis and surgical resection of thyroid in them should be reserved only for relief of compressive symptoms [2,14].

## 4 Conclusion

Metastatic etiology should always be considered in patients who present with a thyroid nodule and have positive history for renal cell carcinoma. It is very difficult to differentiate between primary thryoid carcinoma and secondary metastsis to the thyroid on the basis of pre-operative investigations as well as FN. Even though FNA is a useful tool in thyroid tumor diangosis, it has a high false negativity rate. Meanwhile, immunohistochemistry of the thyroidectomy specimen is more conclusive in establishing the diagnosis. As a result, surgical resection of the thyroid gland should be carried out if FNA proves inconclusive with positive compression symptoms.

## Ethical approval

Since this is a case report, ethical approval was not required from Institutional Review Board.

## Sources of funding

Nil.

## Consent

Consent was obtained from the patient.

## Author contribution

Sherif K Abdelmonim: Primary physician and the surgeon who performed the intervention.

Ammar Habibullah: involved in the care of the patient and data collection.

Ahmad Aldajani: Involved in drafting, writing and editing of the manuscript and abstract.

Mohannad Rajab: Involved in writing, editing and finalizing of the manuscript.

Mohammad Alessa: Involved in editing the manuscript.

Haddad Alkaf: Involved in editing the manuscript.

## Registration of research studies

This is a case report, so registration was not required.

## Guarantor

Sherif K Abdelmonim.

## Provenance and peer review

Not commissioned, externally peer reviewed.

## Declaration of competing interest

There is no conflict of interest.
